# Understanding Disparity: The Role of Social Vulnerability Index and Social Determinants in Orthopedic Surgical Outcomes

**DOI:** 10.26502/josm.511500235

**Published:** 2025-10-31

**Authors:** Kevin Babakhan Vartanian, Tony Eskandar, Vahe Yacoubian, Devendra K. Agrawal

**Affiliations:** 1 Department of Translational Research, College of Osteopathic Medicine of the Pacific, Western University of Health Sciences, Pomona, California, USA; 2 Department of Orthopedic Surgery, Loma Linda University Medical Center, Loma Linda, California, USA

**Keywords:** Area deprivation index, Distressed community index, Home environment, Nutrition disparities, Orthopedic surgery, Social determinants, Social support, Social vulnerability index, Socioeconomic status

## Abstract

While clinical factors are traditionally considered central to orthopedic surgical outcomes, emerging literature highlights the significant impact of social determinants of health. Factors such as socioeconomic status, education, housing and community environment substantially influence patient recovery and post operative outcomes. This review evaluates three validated indices, the Social Vulnerability Index, Area Deprivation Index, Distressed Communities Index, and their association with orthopedic surgical results. Recent studies consistently demonstrate that higher scores in these indices correlate with increased postoperative complications, prolonged hospital stays, higher emergency department visits, elevated readmission rates, and greater mortality risk across orthopedic subspecialties including joint arthroplasty, trauma surgery, and spine surgery. Specifically, the social vulnerability index robustly predicts postoperative complications and increased healthcare resource utilization. Area deprivation index similarly forecasts extended hospitalization and institutional discharge rates, although its link to short-term complications varies. Distressed communities index reliably indicates higher healthcare resource use, though its predictive strength for specific surgical complications remains inconsistent. Despite these correlations, research limitations persist, notably retrospective study designs, inconsistent methodologies and difficulties integrating area level indices with individual patient data. Nevertheless, incorporating social risk assessments into clinical evaluations represents a crucial avenue to reduce disparities and enhance orthopedic patient care.

## 1. Introduction

Orthopedic surgery traditionally emphasizes anatomical precision, surgical technique, and intraoperative decision-making as primary determinants of patient outcomes. However, growing evidence across fields such as trauma, pediatrics and cardiology suggest that social determinants of health can significantly influence patient outcomes [1,2]. Social determinants of health (SDOH) are non-medical factors such as income, education and housing that influence how patients access and recover from care [3]. Social determinants of health (SDOH) have increasingly gained recognition in medicine as key factors shaping patient outcomes beyond traditional clinical variables. Current literature suggests that non-medical elements such as socioeconomic status, housing stability, education, and community environment substantially affect health status, access to care, and recovery trajectories [4]. Healthcare systems are progressively incorporating SDOH into patient assessment tools, risk stratification models, and intervention planning to more accurately predict and address disparities in care [5]. This is not only due to their impact on individual patient outcomes, but more broadly the impact on public and population health. These social factors result in more burden placed on the medical system through increased postoperative complications, longer hospitalizations, and more unplanned acute care [6,7]. By understanding how these factors impact orthopedic outcomes, changes to the method of care provided or a different systematic approach may be utilized for vulnerable populations to allow for more consistent and positive patient outcomes.

Due to the complex, multivariable, and intertwined nature of SDOH, several indices have been developed to quantify community level risk. Among them, the Social Vulnerability Index (SVI), Area Deprivation Index (ADI), and Distressed Communities Index (DCI) have been increasingly used to assess the relationship between social disadvantage and clinical outcomes [8,9]. This review aims to highlight the intersection between these indices and orthopedic outcomes, emphasizing the need to integrate social context into clinical care in orthopedics.

Of the various indices, one emerging tool is the Social Vulnerability Index (SVI) which was developed by the Center for Disease Control (CDC) and the Agency for Toxic Substances and Disease Registry (ATSDR) to identify communities that may be at increased risk of poor outcomes due to underlying social and structural disadvantages [10]. While originally designed for disaster preparedness, SVI has been increasingly used from the healthcare perspective to help predict disparities in medical outcomes [11]. It incorporates fifteen US Census variables grouped into four themes of socioeconomic status, household composition and disability, minority status and language, and finally housing type and transportation [12]. Given the growing application of SVI in predicting surgical risk and therefore planning medical decisions, this review focuses on its role within orthopedic surgery, where its relevance remains underexplored and critical to improving patient outcomes.

The Area Deprivation Index (ADI) is a measure developed to quantify socioeconomic disadvantages at the neighborhood level, capturing elements such as income, education, employment, and housing quality derived from census data. Although initially utilized for epidemiological research and public health planning, ADI has increasingly been applied in healthcare settings to evaluate and predict patient outcomes, highlighting disparities influenced by localized socioeconomic contexts [13,14]. ADI specifically considers factors related to economic stability, housing quality, and educational attainment to offer a comprehensive reflection of community-level deprivation [15]. Given the demonstrated impact of neighborhood conditions on health outcomes and recovery trajectories, this review will also explore the role of ADI within orthopedic surgery, focusing on its potential to guide interventions and improve patient management strategies. Similarly, the Distressed Communities Index (DCI) is another composite measure developed to assess economic hardship and structural disadvantage at the community level. DCI integrates unemployment rates, poverty prevalence, housing vacancies and educational achievement amongst other factors to offer insight into the social environment affecting community health [16]. Originally designed for economic analysis, DCI’s application in healthcare research is expanding to reflect how these factors can influence clinical outcomes.

As healthcare systems place greater emphasis on value-based care with a holistic approach to patient outcomes, understanding the social and environmental context in which patients receive orthopedic treatment becomes increasingly important. Disparities in access, postoperative complications, readmission rates, and long-term functional recovery may be influenced by factors that extend beyond clinical factors. Identifying how these social vulnerabilities impact surgical outcomes can inform targeted interventions and guide policy that accounts for the broader realities faced by patients.

## 2. Methods

To evaluate the relationship between the social determinants of health (SDOH), various indices, and patient outcomes in orthopedic surgery, a comprehensive literature review was conducted. The process involved defining search terms and establishing inclusion and exclusion criteria to focus on studies assessing how community and patient-level social vulnerability influences surgical outcomes. Articles were selected based on their analysis of orthopedic populations and their evaluation of postoperative complications, hospital readmissions, mortality, length of stay, follow-up adherence, or access to care in the context of SVI or SDOH. Priority was given to studies that used validated social indices such as the CDC’s SVI or Area Deprivation Index (ADI), or that investigated the impact of socioeconomic, housing, or demographic variables on orthopedic care. Studies that did not involve orthopedic populations or did not specifically analyze social vulnerability or related health determinants were excluded. The literature search was conducted using PubMed, Scopus. Each search term was created from a combination of three sets of keywords. The first set includes the three indices of SDOH: “social vulnerability index” OR “SVI,” "Area Deprivation Index" OR “ADI,” and "Distressed Communities Index" OR “DCI.” The second set specifies the specialty of orthopedics with the keywords “orthopedic,” “orthopaedic,” “joint arthroplasty,” “joint replacement,” “fracture,” OR “trauma surgery.” The third set specifies complications associated with SDOH, including the keywords “disparities,” “disability,” “failure,” “infection,” “length of stay,” “outcomes,” “readmission,” “re-hospitalization,” OR “rehospitalization.” The review was limited to English-language studies published between 2000 and 2024.

## 3. Results

**Figure 1:**
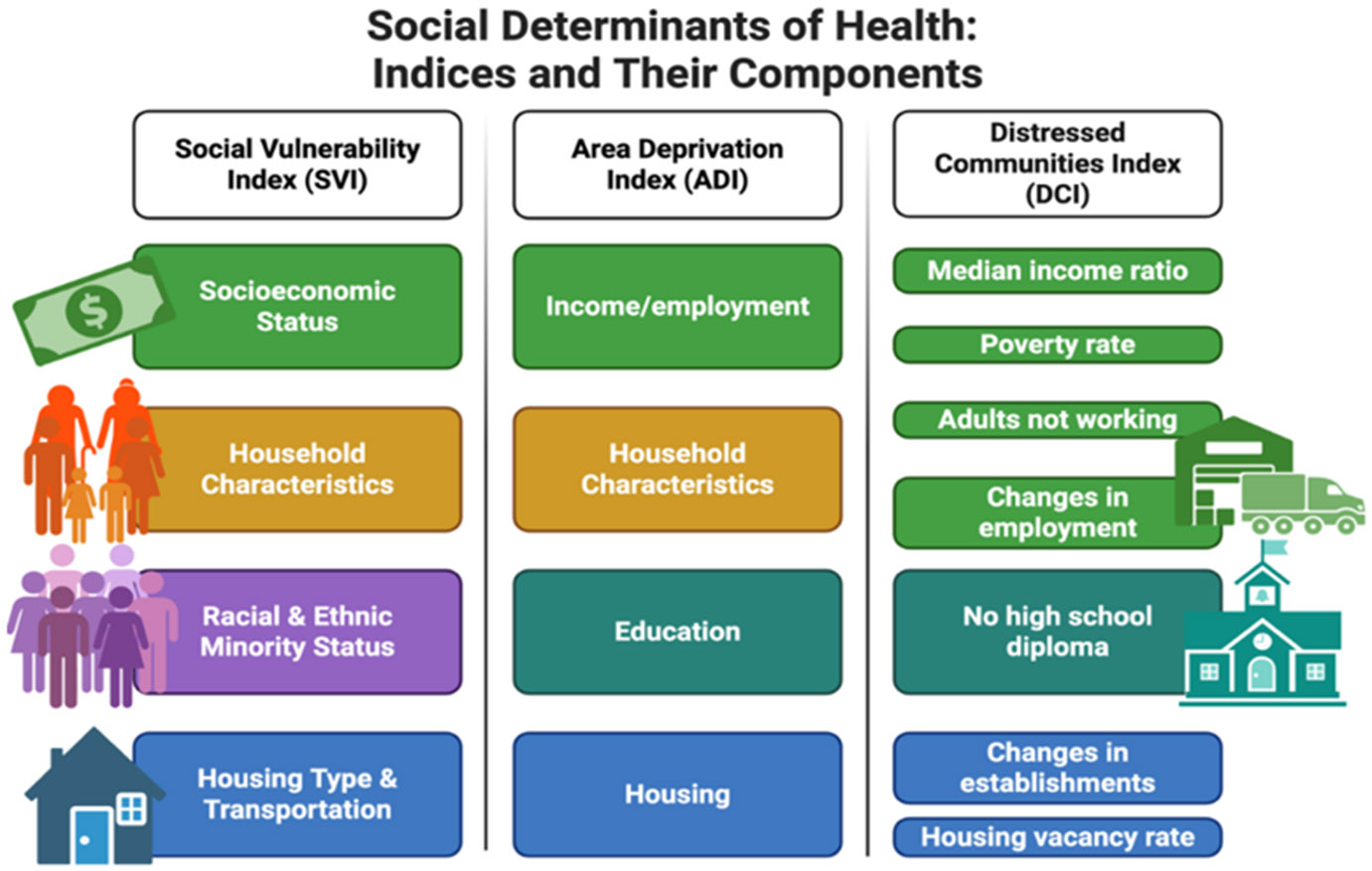
Components of three major indices of social determinants of health (SDOH): Social Vulnerability Index (SVI), Area Deprivation Index (ADI), and Distressed Communities Index (DCI). Overlapping components are color-coded. Both SVI and ADI contain four categories, with SVI uniquely incorporating minority status and ADI including education. DCI comprises seven primarily socioeconomic and employment-related measures, without household or minority-status variables.

There are three major indices of social determinants of health. Each of them will be discussed below with the description on their individual categories (Figure 1).

### 3.1 Social Vulnerability Index (SVI)

Recent studies have examined how SVI and social determinants of health may influence orthopedic outcomes. Emerging evidence indicates that patients from communities with high social vulnerability face elevated risks of adverse orthopedic surgery outcomes. In a statewide registry of over 19,000 total knee arthroplasties (TKA), higher quartiles of the CDC’s Social Vulnerability Index were significantly associated with longer hospital stays, increased rates of 90-day emergency department (ED) visits, and higher readmission rates [17]. Abdelhack et al. [7] conducted a large cross-sectional analysis of over 57,000 patients undergoing surgery across multiple specialties, including orthopedics. The study assessed whether higher SVI scores were associated with increased rates of post-operative complications. They determined that patients with higher SVI scores had greater odds of developing complications such as surgical wound infections, arrhythmias, and heart attacks, particularly in female patients. The strength of these associations diminished after adjusting for comorbidities, suggesting that social vulnerability influences surgical risk in part through baseline health status [7]. Schuster et al. [18] conducted a retrospective cohort study evaluating whether residence in areas with high social vulnerability, measured by the CDC’s Social Vulnerability Index (SVI), was associated with worse outcomes after hip fracture surgery. The study included 464 patients aged 55 and older treated at a single level I trauma center between 2015 and 2020. Patients were grouped by SVI based on residential zip code. Those in high-SVI areas had significantly longer hospital stays and higher 1-year mortality (24% vs. 12.2%, p = 0.005). High SVI was independently associated with increased odds of death post-surgery (OR 1.75, p = 0.045). The authors concluded that social vulnerability is linked to worse surgical outcomes and may help identify at-risk orthopedic patients [18].

Kamalapathy et al. [19] performed a retrospective cohort study using the Mariner Claims Database to evaluate how social determinants of health (SDH) disparities impact outcomes following orthopedic fracture surgery. Among over 200,000 patients undergoing hip or ankle fracture fixation, individuals with educational disparities experienced significantly higher rates of major complications (OR 1.50, p = 0.003) and 90-day readmission (OR 1.38, p < 0.001) after hip surgery. Economic disparities were also linked to increased readmission (OR 1.30, p < 0.001) and 1-year revision risk (OR 1.19, p = 0.018). In ankle fracture patients, both educational and economic disparities were associated with higher rates of infection and readmission [19]. Similarly, a database study of 4,952 TKA patients identified the SVI “household composition and disability” subcomponent as an independent predictor of overall postoperative complications and approximately doubled the odds of any complication [4]. This finding suggests specific social factors such as disabled, single family or elderly household structures are impactful contributors to risk after joint replacement. Higher SVI has also been linked to greater healthcare utilization in the early recovery period. Baxter et al. [5] reported that TJA patients with higher SVI were significantly more likely to return to the hospital via the ED within 90 days of surgery (with an adjusted odds ratio ≈9 for high- vs low-SVI TKA patients) [5]. Despite this increase in acute care, short term functional recovery outcomes showed no statistical difference between socially vulnerable patients and their less vulnerable counterparts. In summary high SVI correlates with higher incidence of complication, readmission, and short-term resource use in orthopedics.

### 3.2 Area Deprivation Index (ADI)

Findings for the Area Deprivation Index, another composite socioeconomic metric, mirror many of the trends observed with SVI. In a Michigan arthroplasty registry analysis, patients in the most deprived neighborhood quartiles by ADI experienced significantly prolonged hospitalizations, more frequent discharges to skilled care facilities, and higher rates of 90-day readmissions and ED visits after TKA [17]. Multivariable modeling confirmed ADI as an independent predictor of these outcomes, indicating that neighborhood deprivation exerts an adverse influence on recovery trajectories following orthopedic procedures. However, the impact of ADI may vary by context and the outcome measure. A single center study of 4,146 primary hip and knee arthroplasties found that higher ADI scores were associated with an increased length of stay, but no rise in 90 day postoperative complications or revisions after controlling for other risk factors [20]. This suggests that deprived areas can extend hospitalization but not inevitably result in short term complications compared to all populations. One study analyzed the impact of SDOH on 477 orthopedic trauma patients using the ADI metric. Results showed 4.8% were lost to follow-up and 28.7% experienced unplanned re-hospitalization, strongly associated with high ADI [21]. Similarly, a study investigating lumbar fusion patients also found an association of high ADI with increased rate of significantly higher rates and odds of respiratory failures and emergency department (ED) visits within 90 days post-surgery [22]. Another study analyzed 3,024 patients to assess the effect of ADI on total joint arthroplasty (TJA). After adjusting for confounding variables, ADI quintiles were not associated with 30-day complications; however, ADI > 47 was linked to increased total complications at 90 days [23]. Beyond orthopedics, the ADI has shown clear prognostic utility. For instance, a general surgery cohort study demonstrated that patients who suffered complications or readmissions had higher ADI scores on average than those without events. In that study, the ADI independently predicted postoperative morbidity (odds ratio ~1.11 per worsening quartile) and its inclusion significantly improved the accuracy of risk models for 30-day complications [24]. These data emphasize that area level deprivation, as quantified by ADI, is a meaningful determinant of surgical outcomes and resource needs.

### 3.3 Distressed Communities Index (DCI)

The Distressed Communities index, reflecting economic hardship at the community level, similarly correlates with variations in orthopedic outcomes. In the large arthroplasty series from Vanderbilt University, higher patient DCI scores were significantly associated with greater inpatient resource utilization, manifested as longer hospital length of stay after total hip or knee arthroplasty [20]. However, the study did not find any significant increase in 90-day postoperative complications, reoperation, or revision rates among patients from highly distressed communities. In a study by Kim et al. [25], no statistically significant differences in the incidence of revision surgeries or infections following total ankle arthroplasty were observed across DCI percentiles [25]. Although patients from highly distressed areas show less functional improvement at two years post op, the difference is not significant, suggesting meaningful recovery is still achievable regardless of community distress. Nonetheless, broader analysis shows community distress can negatively affect surgical outcomes. Another study spanning multiple surgical disciplines found that patients residing in “severely distressed” communities (highest DCI quartile) had higher rates of postoperative complications and short-term mortality even after risk adjustment for clinical factors. A high DCI remained an independent predictor of increased complications post operatively with an odds ratio of 1.1 [16]. Farronato et al found that Patients from distressed communities undergoing primary shoulder arthroplasty exhibited a significantly higher risk of unplanned 90-day readmission compared to those in prosperous areas (OR 1.77, *p* = .045) and demonstrated greater postoperative healthcare utilization, including increased medication prescriptions and follow-up visits [26]. In orthopedic surgery, DCI appears to be most clearly tied to measures of resource utilization (such as prolonged hospitalization), with inconsistent direct effects on complication or readmission rates once other variables are controlled. Nevertheless, the DCI offers a valuable lens for identifying at-risk populations and planning resource allocation, complementing SVI and ADI in capturing the socioeconomic vulnerabilities that shape recovery and outcomes after orthopedic procedures.

## 4. Discussion

Emerging evidence consistently demonstrates that patients from socially vulnerable communities experience worse orthopedic surgical outcomes. High SVI scores correlate with significantly longer hospital stays, higher rates of 90-day emergency department (ED) visits, and increased readmission rates following orthopedic procedures, particularly total knee arthroplasties (TKA) [17]. Similarly, increased social vulnerability significantly predicts postoperative complications, such as wound infections and cardiovascular events, with risks remaining elevated even after adjusting for clinical factors [7]. These findings reflect recent literature, especially the association between elevated SVI and higher mortality rates after hip fracture surgeries [18].

Additionally, the subcomponent “household composition and disability” within SVI emerged as an independent predictor of complications, underscoring specific vulnerabilities such as elderly or disabled households influencing outcomes [4].

Like SVI, the ADI has shown clear associations with orthopedic outcomes. Patients in high-ADI communities experience prolonged hospitalizations, increased discharges to skilled facilities, and greater usage of acute-care resources postoperatively [17]. Recent ADI-focused studies further confirm that higher deprivation notably increases healthcare resource utilization after orthopedic trauma and lumbar spine surgeries [21,22]. While ADI was less consistently predictive of short-term complications across studies, there was a consistent link to increased resource needs, such as extended hospital stays and institutional discharge. This highlights the theme that community deprivation directly impacts orthopedic recovery trajectories.

The Distressed Communities Index (DCI) yielded mixed results. Although studies found that higher DCI scores correlated significantly with increased length of stay and overall healthcare resource utilization after joint arthroplasties, direct associations with 90-day complications or reoperation rates were less evident [20]. This aligns with other recent literature indicating that DCI consistently predicts resource utilization but is less clearly associated with direct postoperative morbidity in orthopedic settings. However, general surgery research confirms DCI's utility in broadly predicting surgical complications, reinforcing its potential as a supplementary measure to better understand socioeconomic influences in orthopedics [16].

While SVI, ADI, and DCI each offer valuable insights into patient risk stratification, inconsistencies exist across orthopedic contexts. Some studies found no independent association between high social vulnerability and short-term complications once covariates were controlled [20]. These findings underscore the complexity of applying broad, area-level indices to individual patients, particularly given that neighborhood-level measures might overlook individual variations in socioeconomic status. This emphasizes the need for integrating patient specific social determinants alongside community level metrics to enhance clinical care.

### 4.1 Social Risk Factors Extend Beyond the Operating Room

Orthopedic outcomes are not determined solely by intraoperative events but are also influenced by patients’ social environments prior to and post operation. Key social determinants of health (SDOH) such as health literacy, caregiver support, housing, and economic instability critically shape recovery trajectories.

Limited health literacy has been linked to worse postoperative outcomes in orthopedic patients. In total knee arthroplasty patients, one study found that suboptimal musculoskeletal health literacy was associated with significantly lower post-surgery functional scores and reduced patient satisfaction [27]. Patients with low health literacy struggle to understand the purpose of discharge instructions and rehabilitation protocols, making them less likely to adhere to these therapies. This ultimately leads to higher complication rates. In fact, limited health literacy may result in increased unplanned healthcare use post orthopedic surgery [27].

**Figure 2:**
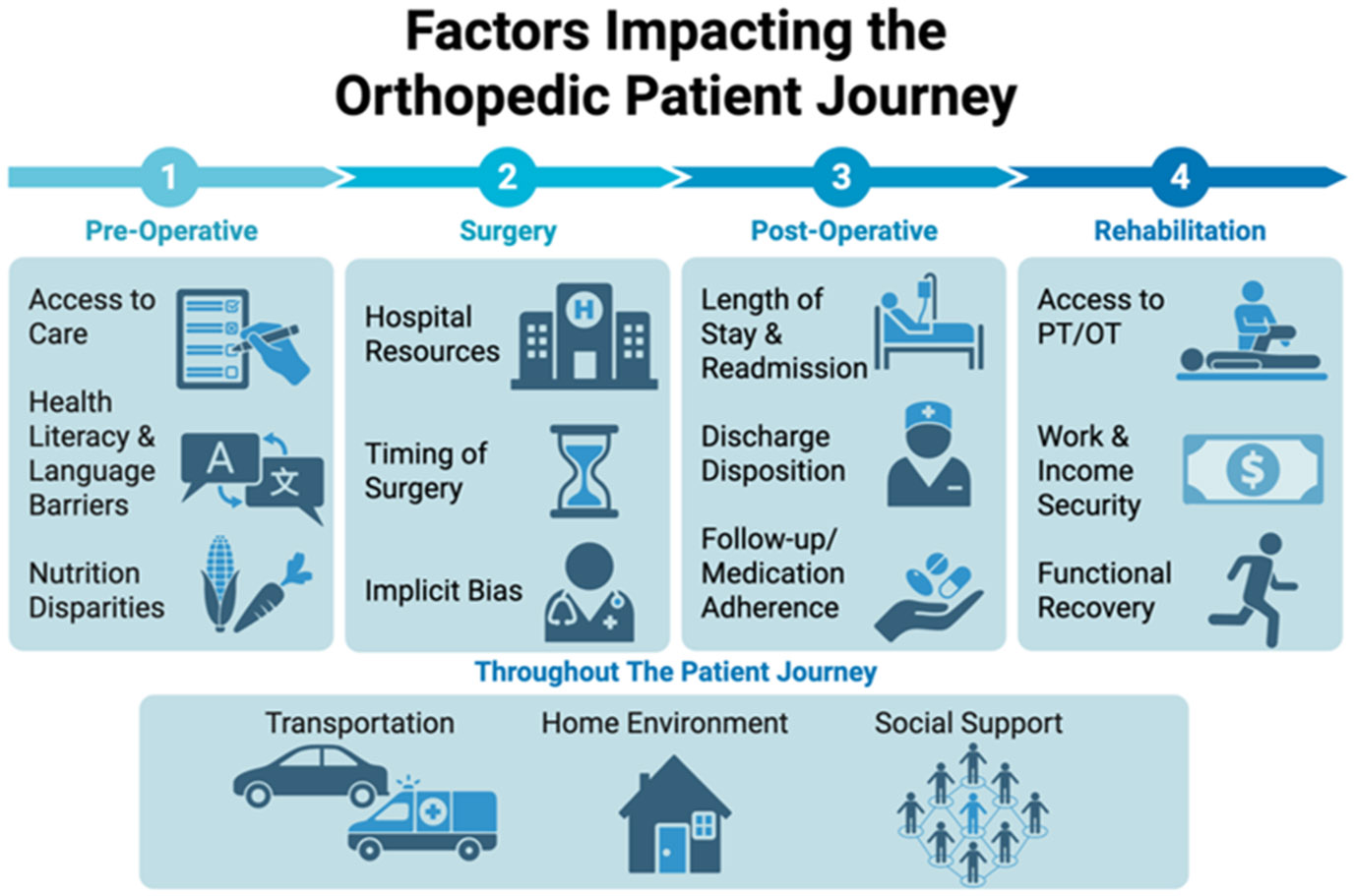
Social factors influence orthopedic patients at multiple stages of care, and their effects may shift over time. Because many of these social determinants are outside the patient’s control, they should be routinely reassessed throughout the treatment course to inform care planning and optimize outcomes.

Strong family or social support, by contrast, has a protective effect on recovery. In older hip fracture patients, those with robust caregiver involvement show markedly better functional recovery and quality of life outcomes. Older adults recovering from hip fracture who report good social support achieve greater improvements in hip motion, strength, and nutrition status compared to those with poor support [28]. Whether family or community-based, social support can accelerate musculoskeletal healing and resilience (Figure 2).

Housing instability exacerbates disparities in postoperative care. Homeless orthopedic patients are more likely to rely on emergency departments and less likely to attend scheduled clinic follow-ups after injury. In a matched cohort study of emergency surgery patients, those without stable housing had half the odds of attending outpatient follow-up and nearly three times the odds of using the ED within 30 days, compared to housed patients [29]. This breakdown in continuity of care means that treatable complications, such as wound care or pain, often go unmanaged until they become emergencies. Not surprisingly, homeless patients also experience higher rates of certain postoperative infections. With hip fractures, after controlling for comorbidities, homelessness has been identified as an independent risk factor for 90-day surgical complications, including higher incidences of surgical site infections and urinary tract infections. Lack of housing after discharge is shown to disrupt recovery and elevates the risk of adverse events [30].

### 4.2 Malnutrition and Economic Instability as Surgical Risk Modifiers

Malnutrition is an often-overlooked driver of complications in orthopedic surgery. Orthopedic trauma patients with nutritional deficits have been shown to suffer roughly twice as many postoperative complications and endure significantly longer hospital stays than well-nourished patients. After stratifying trauma patients by a malnutrition biomarker, one study found malnourished patients to have a higher risk of prolonged length of stay (OR 3.2 for hospital stay >14 days) and double the rate of post-surgical complications during hospitalization [31]. Since major trauma triggers a hypermetabolic inflammatory state, the body begins consuming protein and develops impaired immune function. Consequently, malnourished patients have impaired wound healing, increasing the risk of infections, fracture nonunion, and poor recovery. Emerging evidence suggests that proactive nutritional supplementation in orthopedic trauma patients can improve outcomes by reducing complication rates and muscle wasting [31]. As further studies continue to explore its impact, nutritional status is central to surgical risk stratification.

Economic instability affects access to transportation and adequate care, so this can also hinder rehabilitation and recovery. Patients from socioeconomically deprived neighborhoods tend to have worse adherence to postoperative therapy. Missing therapy not only delays recovery but also increases the risk of joint stiffness and suboptimal functional gains. One cohort study with shoulder instability surgery patients found that those living in areas with high social deprivation (per the Area Deprivation Index) were significantly more likely to skip or miss their physical therapy appointments [32]. Meanwhile, the study also found that patients from more advantaged areas or with adequate insurance coverage are more likely to attend rehab consistently. This highlights how financial and logistical barriers can often limit access to care and transport, directly translating into inferior orthopedic outcomes. Whether as a lack of transport or the inability to afford rehab, economic factors act as risk modifiers that hinder recovery progress.

### 4.3 Fragmentation in the Health System Amplifies Disparity

System-level factors compound these patient-level barriers, particularly for socially vulnerable groups. Patients from disadvantaged backgrounds often struggle to access coordinated postoperative care, leading them to cycle through acute-care settings instead. As noted above, homeless orthopedic patients frequently return to the emergency department in lieu of visiting clinics, resulting in uncoordinated care and higher readmission rates [29]. Even housed patients who are underinsured face similar challenges. Those with Medicaid or no insurance have consistently been shown to be at higher risk of being “lost to follow-up” after orthopedic procedures. In a systematic review of trauma patients, lack of private insurance (Medicaid or uninsured status) was one of the strongest predictors of failing to attend postoperative visits [33]. The consequences of this follow-up gap are serious – missed appointments mean that warning signs of infection, thromboembolism, or poor implant function may go unchecked until an emergency arises. Indeed, broadly speaking, patients on Medicaid experience higher rates of unplanned hospital readmissions after surgery than their privately insured counterparts [34]. Limited outpatient support (such as difficulty finding primary care or transportation) likely contributes to this disparity in readmissions.

These findings make it clear that a patient’s social context fundamentally shapes their orthopedic surgical outcome. Social vulnerability indices – whether evaluating neighborhood disadvantage, health literacy, or access to support – have proven to be potent predictors across arthroplasty, spine, trauma, and sports surgery populations. Traditionally, orthopedic risk assessment has focused on medical factors and technical considerations. However, recognizing that social determinants can substantially influence complications and recovery is crucial to providing equitable, effective care [27,31]. Integrating social risk indices into clinical practice could enable surgeons and care teams to identify high-risk patients and intervene proactively. For example, patients flagged for high social vulnerability might benefit from enhanced discharge planning (e.g. arranging home health or nutritional support), closer postoperative monitoring, or community resource referrals. On a policy level, incorporating SDOH metrics like the Social Vulnerability Index into risk-adjusted payment models could incentivize hospitals to invest in services that address social needs (such as patient navigators or transportation programs). The evidence to date supports that when healthcare systems account for social risk factors – rather than treating all patients as socially equal – disparities in orthopedic surgical outcomes can be reduced [31,32]. In sum, optimizing orthopedic care in the 21st century will require looking beyond the operating room to the social conditions that fundamentally determine patients’ healing and long-term recovery.

### 4.4 Challenges

Despite the growing recognition of social determinants of health as critical predictors of orthopedic surgical outcomes, several important challenges exist within current research. A primary limitation involves the retrospective nature and observational designs utilized in most studies, which inherently limit the ability to establish causality. Although significant associations between indices such as SVI, ADI, and DCI and surgical outcomes have been consistently identified, these correlations do not definitively confirm that social vulnerabilities directly cause adverse outcomes. Prospective, interventional studies are notably scarce, leaving unanswered the crucial question of whether targeted pre- or postoperative interventions based on these indices can meaningfully reduce complication rates, readmissions, or improve functional outcomes [23].

Another challenge is methodological inconsistency across studies, complicating comparisons and limiting generalizability. Researchers have applied different thresholds to categorize patients in different sets of social vulnerability, with certain researchers using quartiles, others utilizing median splits, and some simply categorizing into a “high” or “low risk” group. Furthermore, variability exists regarding which outcomes are assessed, from complication rates to length of stay. While this analysis is helpful, the substantial heterogeneity introduced can limit the strong predictive power of these indices. Additionally, while SVI, ADI, and DCI all measure aspects of socioeconomic disadvantage, the differing variable composition means that they are not exactly interchangeable. However, few studies have directly compared these indices within the same population or study sample to help ascertain a better understanding of how these differences amalgamate. In the future, more homogenous study designs utilizing prospective research designs will help show causality as well as more concrete evidence behind the influence of SDOH on orthopedic outcomes.

## 5. Conclusion

The integration of social determinants of health into orthopedic research is essential to improving surgical outcomes and addressing persistent disparities. This article highlights that indices such as the Social Vulnerability Index (SVI), Area Deprivation Index (ADI), and Distressed Communities Index (DCI) consistently correlate with increased postoperative complications, prolonged hospital stays, and elevated healthcare resource utilization across multiple orthopedic subspecialties. While these indices vary in predictive strength depending on the population and outcome studied, they collectively highlight the critical influence of socioeconomic status on recovery trajectories. Incorporating these metrics into clinical risk assessment, planning, and policy development offers a promising future toward more equitable orthopedic care. However, to move from correlation to causation and actionable change, future research must focus on prospective studies, standardization of methodologies, and head-to-head comparisons of existing indices. Adopting this more holistic view of the patient that accounts for both clinical and social complexity will be key to advancing patient centered care and closing the gap in orthopedic health disparities.

Integrating these indices into routine clinical assessments could inform targeted interventions such as preoperative risk stratification, tailored discharge planning, and allocation of additional postoperative resources. Recognizing patients at higher social risk could facilitate early intervention strategies, potentially reducing avoidable complications and improving functional outcomes. Additionally, policy frameworks that incorporate social vulnerability metrics into performance measures and reimbursement structures may incentivize healthcare providers to proactively observe and manage socioeconomic risk factors. Further research is needed to evaluate whether targeted interventions based on social indices meaningfully impact outcomes and reduce healthcare disparities. Exploring which social factors are economically, socially and clinically viable to focus on will help push towards system wide changes. By systematically addressing both clinical and social determinants, orthopedic surgery can advance towards a more comprehensive model of patient care that not only improves surgical results but also fosters equity and better health outcomes for vulnerable patient populations.
